# Corrosion resistance of monolayer hexagonal boron nitride on copper

**DOI:** 10.1038/srep42139

**Published:** 2017-02-13

**Authors:** F. Mahvash, S. Eissa, T. Bordjiba, A. C. Tavares, T. Szkopek, M. Siaj

**Affiliations:** 1 Department of Chemistry, Université du Québec à Montréal, Montréal, Quebec, H3C 3P8, Canada; 2 Department of Electrical and Computer Engineering, McGill University, Montréal, Quebec, H3A 2A7, Canada; 3 Institut national de la recherche scientifique (INRS), Centre Énergie, Matériaux et Télécommunications, Varennes, Québec, J3X 1S2, Canada; 4 Département de génies des procédés, Laboratoire de Génie Electrique de Guelma. Université de Guelma, B.P 401 Guelma, Algérie

## Abstract

Hexagonal boron nitride (hBN) is a layered material with high thermal and chemical stability ideal for ultrathin corrosion resistant coatings. Here, we report the corrosion resistance of Cu with hBN grown by chemical vapor deposition (CVD). Cyclic voltammetry measurements reveal that hBN layers inhibit Cu corrosion and oxygen reduction. We find that CVD grown hBN reduces the Cu corrosion rate by one order of magnitude compared to bare Cu, suggesting that this ultrathin layer can be employed as an atomically thin corrosion-inhibition coating.

Hexagonal boron nitride (hBN) is a layered, wide bandgap material that has attracted much interest in nanoelectronics. In particular, hBN has found use as a high-quality insulating dielectric layer in ultra-high mobility graphene devices, 2-dimensional heterostructures and tunneling devices^1^,^2^,^3^,^4^. Previous work has shown that hBN has high thermal and chemical stability^5^,^6^, and monolayer hBN is impermeable to oxygen diffusion even at high temperatures in an oxidizing atmosphere and air^7^,^8^. In other studies, the electrochemical analyses reveal that the hBN nanosheet coating (15–20 layers) can increase open circuit potential and suppress the oxidation of the underlying Cu foil^9^,^10^. These results together suggest hBN may be suitable as a corrosion inhibiting coating, where high quality, continuous hBN layers without pinholes and tears are required for such an application.

The suitability of an atomic monolayer for inhibiting corrosion has been established in studies of graphene, a material sharing the same layered hexagonal crystal structure as hBN. Graphene layers on Ni and Cu can serve as barriers to electrochemical corrosion in aqueous media^11^. An increase in the resistance to electrochemical degradation exceeding one order of magnitude has been observed in graphene-coated Cu^12^. In another study, the corrosion-inhibiting behavior of single-layer and multilayer graphene was quantitatively investigated^13^, where it was found that Cu foils with a graphene monolayer experience a ~7 fold reduction in corrosion rate as compared to bare Cu. The electrically insulating nature of hBN, in contrast with the semi-metallic nature of graphene, is anticipated to favor hBN as a corrosion inhibiting layer by suppressing electron transfer even in a long term. Recently, the long-term barrier characteristics of hBN and graphene have been investigated theoretically and experimentally by exposing them to an ambient environment for 160 days^14^.

We report here quantitative measurements of monolayer hBN as a Cu corrosion inhibitor by use of cyclic voltammetry, Tafel analysis and electrochemical impedance spectroscopy (EIS) in a 0.1 M NaOH solution. We have observed that monolayer hBN grown by chemical vapor deposition (CVD) can protect the underlying Cu substrate from oxidation, reducing the corrosion rate by one order of magnitude.

## Results

The hBN monolayers were grown on 25 μm thick Cu foils at low-pressure (600 mTorr) and high furnace temperature (1000 °C) using a CVD method similar to that previously reported^15^. The precursor was ammonia borane (NH_3_−BH_3_, Sigma Aldrich). Further details concerning hBN growth are provided in the Supplementary Data. Upon the completion of growth, a portion of the CVD grown hBN on Cu samples was directly used in electrochemical measurements and the other portions were characterized by Raman spectroscopy, atomic force microcopy (AFM), optical reflection microscopy and transmission electron microcopy (TEM) and selected-area electron diffraction (SAED).

**Figure 1:**
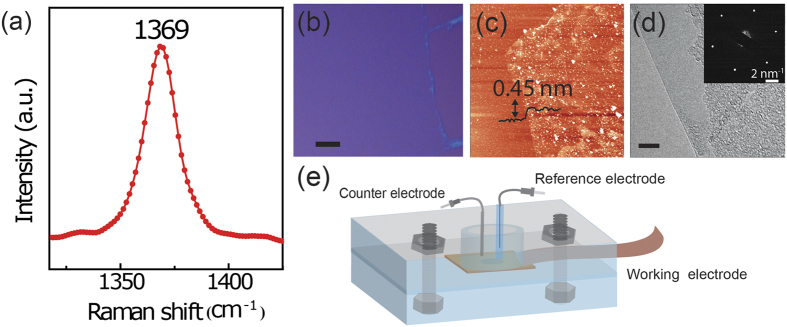
(**a**) Raman Stokes spectrum with a Stokes peak at 1369 cm-1 corresponding to an hBN monolayer. (**b**) an optical reflection image of a uniform CVD-grown hBN monolayer transferred onto a 300 nm thick SiO_2_/Si substrate, the scale bar is 20 µm. (**c**) an AFM image of the hBN monolayer with cross-sectional height profile analysis showing the step height of hBN on SiO_2_ to be 0.45 nm. (**d**) TEM image of a suspended hBN monolayer edge over Cu foil, the scale bar is 5 nm and the inset shows the selected area electron diffraction pattern with the expected hexagonal lattice structure of hBN monolayer (**e**) Schematic of the custom built PTFE electrochemical cell. Bare Cu or hBN-Cu samples were clasped in the PTFE cell with a 0.07 cm^2^ area opening to the electrolytic solution.

For the purpose of Raman spectroscopy, CVD grown hBN monolayers were transferred from Cu to a 300 nm thick SiO_2_/Si substrate by a polymer handle method. A Raman Stokes spectrum of a representative sample is shown in Fig. 1a. A single Stokes peak at 1369 cm^−1^ was observed by fitting to a single Lorentzian peak, in accordance with that reported for monolayer hBN prepared by exfoliation of a single crystal of hBN^16^. Figure 1b displays an optical reflection image of a uniform monolayer transferred to a Si substrate with 300 nm thick SiO_2_ for enhanced visible reflection contrast of 2–3% in the visible region [16]. An AFM image of the same sample is shown in Fig. 1c. The AFM image shows that the films were uniform with some PMMA residue originating from the transfer process. The thickness of the hBN layer is less than 0.45 nm, which is slightly larger than the layer spacing of bulk hBN and in good agreement with the expected thickness measured by AFM for monolayer hBN. TEM and SAED was performed on the edge of suspended pristine hBN layers by using a partial etch of the Cu substrate [17]. A bright-field TEM image illustrated in Fig. 1d reveals a continuous and transparent layer and the SAED measurement shown in the inset of Fig. 1d gives the expected hexagonal lattice structure of hBN monolayer.

Electrochemical measurements were conducted with a standard three-electrode configuration in a custom built polytetrafluoroethylene (PTFE) cell filled with a 0.1 M NaOH (Sigma Aldrich) solution at room temperature. A schematic of the PTFE cell is illustrated in Fig. 1e. Two types of samples were measured: bare Cu foils and Cu foils with an hBN monolayer grown directly on the surface by CVD and designated hBN-Cu. In all measurements, the Cu or hBN-Cu foils with a 0.07 cm^2^ area were used as the working electrode, a Pt wire was used as a counter electrode and Ag/AgCl was used as a reference electrode. All electrochemical measurements were carried out using a potentiostat/galvanostat with a voltage sweep rate of 20 mV/s.

**Figure 2:**
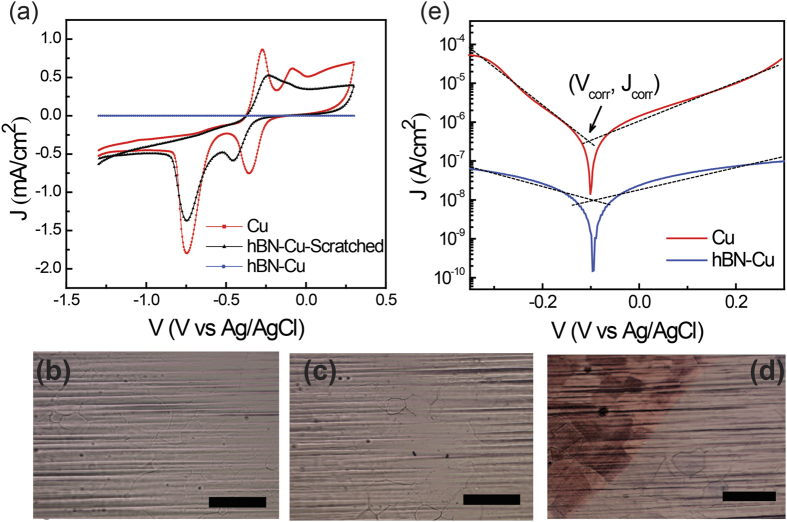
(**a**) CV measurements for a 0.07 cm^2^ area bare Cu (red), hBN-Cu (blue) and scratched hBN Cu (black) in a 0.1 M NaOH solution. Optical microscope images of hBN-Cu before (**b**) and after (**c**) 30 consecutive CV sweeps. (**d**) Optical microscope image of scratched hBN-Cu after 30 CV sweeps. Scale bar is 100 μm in (**b**), (**c**) and (**d**). (**e**) Tafel plots of Cu (red) and hBN-Cu (blue) samples, with linear fits of cathodic and anodic curves (dashed line) giving the intersection at a potential *Vcorr* and current density *Jcorr*.

Cyclic voltammetry (CV) was first used to investigate the surface electrochemistry of the Cu and hBN-Cu samples. Figure 2a illustrates the CV measurements of bare Cu, hBN-Cu and hBN-Cu with a purposefully introduced scratch, in a 0.1 M NaOH electrolyte solution. Two anodic current peaks and two cathodic current peaks were observed for bare Cu due to two Cu redox reactions^17^. The anodic peaks at cell potentials of −0.3 V and −0.1 V are due to the forward reactions and the cathodic peaks at −0.4 V and −0.75 V are attributed to the reverse reactions. The two principle Cu redox reactions are,


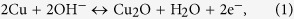



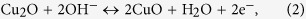


However, no peaks were found for hBN-Cu samples. The anodic and cathodic currents were suppressed by over three orders of magnitude in comparison with bare Cu samples. There were no measureable cathodic or anodic peaks signaling Cu oxidation after 30 consecutive CV sweeps. These results indicate that hBN is effective in protecting Cu from oxidation and isolating the Cu from the electrolytic solution. The same hBN-Cu sample was mechanically scratched to expose bare Cu and the CV measurements were repeated. As shown in Fig. 2a, the anodic and cathodic peaks of Cu reappeared in the hBN-Cu sample with a mechanically scratched surface.

Optical images were taken of the sample surfaces before and after CV measurements. Figure 2b and c show the hBN-Cu sample before and after 30 CV sweeps, respectively. The hBN-Cu surface was unchanged after 30 consecutive CV sweeps. However, the oxidation of Cu was directly observed after 30 CV sweeps in the areas of Cu exposed by a mechanical scratch, as clearly seen in the optical image of Fig. 2d. Notably, the striations in the Cu foil visible in Fig. 2b–d that result from the cold roll manufacturing process of thin foils do not inhibit the formation of a continuous hBN layer over the Cu foils. The CVD hBN forms a conformal layer over the Cu surface.

We determined the corrosion rate (CR) for our samples. The corrosion of Cu includes both oxidation and reduction reactions, and the CR is accordingly determined by the kinetics of both anodic and cathodic reactions^18^. For uniform corrosion, the CR is related directly to the corrosion current density, *J*_corr_ through the simple relation^19^:


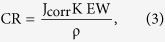


where *K* = 3272 mm/A·cm·year is the corrosion rate constant, and *EW* and *ρ* are the equivalent weight and mass density of the corroding species, respectively. The current density under an electrochemical potential difference *V* is modeled by the Butler-Volmer equation, in which the kinetics of the electron-transfer reaction are assumed to dominate^20^:


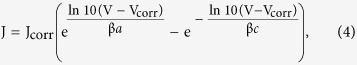


where *V*_*corr*_ is the potential at which the rate of anodic and cathodic processes are equal, and *β*_*a*_ and *β*_*c*_ are the anodic and cathodic Tafel constants, respectively.

A Tafel analysis of the logarithm of current density *J* versus potential *V* for both reactions was used to infer the corrosion current *J*_corr._, as shown in Fig. 2e. The intersection of the linear fits to anodic and cathodic branches gives an estimate of *J*_*corr*_ = (1.22 ± 0.22) × 10^−8^ A/cm^2^ for hBN-Cu and *J*_*corr*_ = (3.08 ± 0.03) × 10^−7^ A/cm^2^ for bare Cu. These values are the average values over three samples ± standard deviation. The corrosion current of hBN-Cu is almost one order of magnitude less than that of bare Cu. Noting the mass density *ρ* = 8.94 g/cm^3^ and equivalent weight *EW* = 31.7 g of Cu, the extracted average CR for Cu was (3.57 ± 0.03) × 10^−3 ^mm/year, which is in accord with literature values^13^,^21^. A CR of (1.41 ± 0.25) × 10^−4 ^mm/year was obtained for hBN-Cu, which is approximately one order of magnitude less than that of bare Cu. This analysis demonstrates that an hBN monolayer acts as a Cu corrosion inhibitor with better performance than graphene and other corrosion inhibiting layers^12^,^22^,^23^. This behavior could be a result of insulating nature of hBN which suppresses the electrochemical reactions.

**Figure 3:**
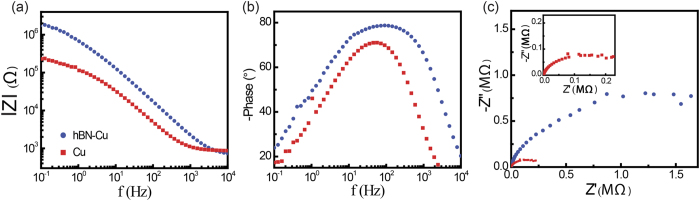
EIS results of bare Cu and hBN-Cu in a 0.1 M NaOH electrolyte solution upon application of a 10 mV sinusoidal AC potential and a DC potential of 0.1 V versus Ag/AgCl to the working electrode. (**a**) Bode plot of impedance magnitude of Cu (red) and hBN-Cu (blue) samples. (**b**) A Bode phase plot of Cu (red) and hBN-Cu (blue) samples. (**c**) Nyquist impedance plots of Cu (red) and hBN-Cu (blue) sample. Inset, Nyquist impedance plot of Cu.

The corrosion inhibition in hBN-Cu was further validated by EIS. The impedance from electrolyte to electrode versus frequency was measured by application of a 10 mV sinusoidal AC potential and a DC potential of 0.1 V versus Ag/AgCl to the working electrode, over a frequency range of 0.1 Hz to 10 kHz. EIS results of bare Cu and hBN-Cu in a 0.1 M NaOH electrolyte under identical experimental condition are presented in Fig. 3. The most prominent difference between hBN-Cu and Cu is the increased electrochemical impedance of hBN-Cu versus Cu, particularly at low frequencies. A higher impedance at low frequencies is most naturally explained by an increase in charge transfer resistance as a result of the hBN layer. As shown in Fig. 3a and b, the impedance of hBN-Cu and Cu drop as frequency increases. Figure 3b shows a broad phase angle for hBN-Cu which suggests the existence of two overlapped time constants. The electrochemical impedance of graphene coated Cu typically adheres to a Randle-Warburg circuit model^13^. However, the Nyquist plots of both hBN-Cu and Cu samples (Fig. 3c) do not display the expected impedance semicircle at high frequency and the linear region with 45° slope at low frequency. Qualitatively, the impedance drops at high frequency as the capacitive impedance of the electrolyte-electrode interfaces, including the hBN layer itself, become more effective at shunting the charge transfer resistances. A more complex circuit model is required to accurately model the measured EIS results, and further studies are required in this regard.

In conclusion, the results of this investigation show that CVD grown hBN monolayers can serve as corrosion-inhibiting layers. We found that CVD grown hBN reduces the Cu corrosion rate by one order of magnitude compared to bare Cu. The practical adoption of hBN monolayers as atomically thin anti-corrosion coatings entails further development of facile synthesis techniques for large area hBN without any pinholes. Moreover, thorough investigation of pinhole and tear density over areas exceeding 1 cm^2^ and further investigations of mechanical stability are required.

## Methods

The Cu foils (Alfa Aesar) were washed with warm acetic acid (60 °C) for 15 minutes to remove the oxide layer followed by rinsing with deionized water and isopropanol alcohol (IPA). The cleaned Cu foils were kept in a beaker of IPA for electrochemical measurements (bare Cu sample) as well as for CVD growth. The hBN transfer to a 300 nm thick SiO_2_/Si was done by use of polymethyl methacrylate (MicroChem) handle and etching of Cu in a 0.1 M ammonium persulphate (Sigma Aldrich) solution. Raman spectroscopy (Renishaw inVia) was performed with 514.5 nm laser excitation and grating of 1800 l/mm at a power of 20 mW. TEM was carried out on a JEOL JEM-2100F at 200 kV. AFM image was acquired in air using silicon cantilevers operated in Bruker’s ScanAsyst. TEM and SAED were carried out on a JEOL JEM-2100F at 200 kV. All electrochemical measurements were carried out in a PTFE cell by use of an Autolab PGSTAT302N potentiostat with NOVA software version 1.9. The Ag/AgCl was purchased from BASi.

## Additional Information

**How to cite this article**: Mahvash, F. *et al*. Corrosion resistance of monolayer hexagonal boron nitride on copper. *Sci. Rep.* **7**, 42139; doi: 10.1038/srep42139 (2017).

**Publisher's note:** Springer Nature remains neutral with regard to jurisdictional claims in published maps and institutional affiliations.

## References

[b1] Mayorov A. S. . Micrometer-Scale Ballistic Transport in Encapsulated Graphene at Room Temperature. Nano Lett. 11, 2396–2399 (2011). 21574627 10.1021/nl200758b

[b2] Dean C. R. . Boron nitride substrates for high-quality graphene electronics. Nat. Nanotechnol. 5, 722–726 (2010). 20729834 10.1038/nnano.2010.172

[b3] Wang X. & Xia F. Van der Waals heterostructures: Stacked 2D materials shed light. Nat. Mater. 14, 264–265 (2015). 25643031 10.1038/nmat4218

[b4] Britnell L. . Field-Effect Tunneling Transistor Based on Vertical Graphene Heterostructures. Science 335, 947–950 (2012). 22300848 10.1126/science.1218461

[b5] Haubner R., Wilhelm M., Weissenbacher R. & Lux B. In High Performance Non-Oxide Ceramics II (ed. Jansen P. D. M.) 1–45 (Springer Berlin Heidelberg, 2002).

[b6] Li L. H., Cervenka J., Watanabe K., Taniguchi T. & Chen Y. Strong Oxidation Resistance of Atomically Thin Boron Nitride Nanosheets. ACS Nano 8, 1457–1462 (2014). 24400990 10.1021/nn500059s

[b7] Liu Z. . Ultrathin high-temperature oxidation-resistant coatings of hexagonal boron nitride. Nat. Commun. 4, 2541 (2013). 24092019 10.1038/ncomms3541

[b8] Li X., Yin J., Zhou J. & Guo W. Large area hexagonal boron nitride monolayer as efficient atomically thick insulating coating against friction and oxidation. Nanotechnology 25, 105701 (2014). 24532053 10.1088/0957-4484/25/10/105701

[b9] Li L. H., Xing T., Chen Y. & Jones R. Boron Nitride Nanosheets for Metal Protection. Adv. Mater. Interfaces 1, 1300132 (2014).

[b10] Zhang J., Yang Y. & Lou J. Investigation of hexagonal boron nitride as an atomically thin corrosion passivation coating in aqueous solution. Nanotechnology 27, 364004 (2016). 27483462 10.1088/0957-4484/27/36/364004

[b11] Kirkland N. T., Schiller T., Medhekar N. & Birbilis N. Exploring graphene as a corrosion protection barrier. Corros. Sci. 56, 1–4 (2012).

[b12] Singh Raman R. K. . Protecting copper from electrochemical degradation by graphene coating. Carbon 50, 4040–4045 (2012).

[b13] Prasai D., Tuberquia J. C., Harl R. R., Jennings G. K. & Bolotin K. I. Graphene: Corrosion-Inhibiting Coating. ACS Nano 6, 1102–1108 (2012). 22299572 10.1021/nn203507y

[b14] Shen L. . A long-term corrosion barrier with an insulating boron nitride monolayer. J. Mater. Chem. A 4, 5044–5050 (2016).

[b15] Mahvash F., Paradis E., Drouin D., Szkopek T. & Siaj M. Space-Charge Limited Transport in Large-Area Monolayer Hexagonal Boron Nitride. Nano Lett. 15, 2263–2268 (2015). 25730309 10.1021/nl504197c

[b16] Gorbachev R. V. . Hunting for Monolayer Boron Nitride: Optical and Raman Signatures. Small 7, 465–468 (2011). 21360804 10.1002/smll.201001628

[b17] Wan Y. . Corrosion behavior of copper at elevated temperature. Int J Electrochem Sci 7, 7902–7914 (2012).

[b18] Ahmad Z. Principles of Corrosion Engineering and Corrosion Control. (Butterworth-Heinemann, 2006).

[b19] Gamry Instruments. Application Note: Electrochemical Corrosion Measurements. Available at: http://www.gamry.com/application-notes/corrosion-coatings/basics-of-electrochemical-corrosion-measurements/ (Accessed: 27th July 2015) (1990).

[b20] Metrohm Autolab. Applications notes. Available at: http://www.ecochemie.nl/Applications/ (Accessed: 27th July 2015) (2010).

[b21] Al-Dokheily M. E., Kredy H. M. & Al-Jabery R. N. Inhibition of Copper Corrosion in H2SO4, NaCl and NaOH Solutions by Citrullus colocynthis Fruits Extract. J. Nat. Sci. Res. 4, 60–73 (2014).

[b22] Merisalu M. . Graphene–polypyrrole thin hybrid corrosion resistant coatings for copper. Synth. Met. 200, 16–23 (2015).

[b23] Appa Rao B. V., Yakub Iqbal M. & Sreedhar B. Self-assembled monolayer of 2-(octadecylthio)benzothiazole for corrosion protection of copper. Corros. Sci. 51, 1441–1452 (2009).

